# Amelioration of Cancer Cachexia by *Dalbergia odorifera* Extract Through AKT Signaling Pathway Regulation

**DOI:** 10.3390/nu16213671

**Published:** 2024-10-28

**Authors:** Phuong T. Ho, Eulyong Park, Quynh Xuan Thi Luong, Meutia Diva Hakim, Phuong T. Hoang, Thuy T. B. Vo, Kantawong Kawalin, Hee Kang, Taek-Kyun Lee, Sukchan Lee

**Affiliations:** 1 Department of Integrative Biotechnology, Sungkyunkwan University, Suwon 16419, Republic of Korea; hophuongk59sinhhoc@gmail.com (P.T.H.); quynh.ltx2017@gmail.com (Q.X.T.L.); meutia.divahakim@gmail.com (M.D.H.); hoangphuong06cs@gmail.com (P.T.H.); bichthuy251188@gmail.com (T.T.B.V.); aomkawarin@gmail.com (K.K.); 2 R&D Center, Easthill Corporation, Suwon 16642, Republic of Korea; eulyongpark@daum.net; 3 Humanitas College, Kyung Hee University, 1732 Deogyeongdae-ro, Yongin 17104, Republic of Korea; shehee@khu.ac.kr; 4 Ecological Risk Research Department, Korea Institute of Ocean Science & Technology, Geoje 53201, Republic of Korea

**Keywords:** *Dalbergia odorifer* extract, cancer cachexia, muscle atrophy, adipose wasting, AKT

## Abstract

**Background/Objectives:** Cancer cachexia is a multifactorial syndrome characterized by the progressive loss of skeletal muscle mass and adipose tissue. *Dalbergia odorifer* is widely used in traditional medicine in Korea and China to treat various diseases. However, its exact role and underlying mechanism in regulating cancer cachexia have not been elucidated yet. This research was conducted to investigate the effect of D. odorifer extract (DOE) in preventing the development of cancer-induced cachexia symptoms and figure out the relevant mechanisms. **Methods:** A cancer cachexia model was established in Balb/c mice using the CT26 colon carcinoma cell line. To evaluate the anti-cachexia effect of *Dalbergia odorifer* extract (DOE), CT26-bearing mice were orally administered with DOE at concentrations of 50 and 100 mg/kg BW for 14 days. C2C12 myotubes and 3T3L1 adipocytes were treated with 80% CT26 conditioned medium, DOE, and wortmannin, a particular AKT inhibitor to determine the influence of DOE in the AKT signaling pathway. Mice body weight, food intake, myofiber cross-sectional area, adipocyte size, myotube diameter, lipid accumulation, and relevant gene expression were analyzed. **Results:** The oral administration of DOE at doses of 50 and 100 mg/kg body weight to CT26 tumor-bearing mice resulted in a significant reduction in body weight loss, an increase in food intake, and a decrease in serum glycerol levels. Furthermore, DOE treatment led to an increase in muscle mass, larger muscle fiber diameter, and elevated expression levels of MyH2 and Igf1, while simultaneously reducing the expression of Atrogin1 and MuRF1. DOE also attenuated adipose tissue wasting, as evidenced by increased epididymal fat mass, enlarged adipocyte size, and upregulated Pparγ expression, alongside a reduction in Ucp1 and IL6 levels. In cachectic C2C12 myotubes and 3T3-L1 adipocytes induced by the CT26 conditioned medium, DOE significantly inhibited muscle wasting and lipolysis by activating the AKT signaling pathway. The treatment of wortmannin, a specific AKT inhibitor, effectively neutralized DOE’s impact on the AKT pathway, myotube diameter, and lipid accumulation. **Conclusions:** DOE ameliorates cancer cachexia through the expression of genes involved in protein synthesis and lipogenesis, while suppressing those related to protein degradation, suggesting its potential as a plant-derived therapeutic agent in combating cancer cachexia.

## 1. Introduction

Cancer cachexia is a multifactorial syndrome occurring in many advanced cancers, which is characterized by the ongoing loss of muscle mass (with or without fat loss) that cannot be completely reversed by conventional nutritional support and leads to progressive functional impairments [1,2]. In patients with cachexia, body weight losses account for >5% in <12 months underlying illness or a body mass index of <20 kg/m^2^ when the ongoing weight loss is >2% [3]. Moreover, low muscle mass, along with decreased muscle strength, fatigue, anorexia, and increased systemic inflammatory responses, is also observed [4]. The cachectic state is highly associated with a poor prognosis, reduced quality of life, increased treatment-related toxicities, and reduced survival rate [5], making it a recent focus of cancer treatment and management [6]. Up to 80% of patients with advanced cancers suffer from cachexia, and at least 20% of cancer mortality is caused by cachexia [7,8].

In normal physiological conditions, adult muscle mass remains constant because of a balance in protein synthesis and degradation. During tumor progression, this balance is disrupted, with accelerated protein degradation and reduced protein synthesis during tumor progression [9]. An increased expression of genes regulating the ubiquitin–proteasome pathway such as MuRF1 and Atrogin1 in the muscles of patients with cancer cachexia has been commonly observed [10]. In adipose tissue, cell morphology and its function undergo extensive remodeling during cachexia [5]. Adipose tissue loss in cancer cachexia is thought to be due to increases in lipolytic activity and reductions in adipogenesis and lipogenesis [11].

The balance in muscle growth and adipose mass is heavily dependent on the AKT signaling pathway [12,13]. In skeletal muscles, AKT phosphorylation by growth factors and nutrients promotes the activation of downstream targets involved in the stimulation of protein translation and inhibition of protein breakdown through PI3K-dependent processes [14]. However, during cancer cachexia, AKT pathway activity decreases, leading to the activation of the FoxO3α transcription factor and expression of E3 ubiquitin ligases, which induces muscle atrophy [15,16]. The activation of AKT signaling in adipose tissue also regulates the expression of adipogenic transcription factors relating to lipid metabolism, which inhibits lipolysis, and promotes lipid storage and cell differentiation [17]; however, this process is impaired during cachexia, resulting in adipose wasting [18].

*Dalbergia odorifer* is a valuable rosewood tree that has been broadly used in traditional medicine in Korea and China for treating cardiovascular diseases, blood disorders, ischemia, and relieving pain [19]. In addition, its plant extract shows various biological activities, such as anti-inflammatory, antioxidant, antiplatelet aggregation, angiogenic, antimicrobial, and vasodilatory effects [20]. However, the anticancer cachexia efficacy of this extract and its underlying mechanisms have not been elucidated.

This study aimed to investigate whether *D. odorifer* extract (DOE) could inhibit the development of cancer-induced cachexia symptoms in Balb/c mice bearing CT-26 colon carcinoma, including body weight, skeletal muscle, fat mass loss, and anorexia. Moreover, C2C12 myoblasts and 3T3L1 preadipocytes will be used to determine the effects and mechanism of this plant extract on its ability to reduce muscle atrophy and lipolysis.

## 2. Materials and Methods

### 2.1. Reagents and Antibodies

Antibodies against Atrogin1 (1:500) (A3193), FOXO3A (1:1000) (A9270), *p*-FOXO3A-S253 (1:1000) (AP0684), and UCP1 (1:1000) (A5857) were purchased from Abclonal (Woburn, MA, USA). Antibodies against AKT (1:1000) (9272S), P-AKT-S473 (1:1000) (9271S), goat anti-rabbit HRP-linked (1:5000), and horse anti-mouse HRP-linked (1:5000) were obtained from Cell Signaling Technology (Danvers, MA, USA). Antibodies against MYH2 (1:1000) (PA5-119292) and PPARγ (1:1000) (PA3-821A) were obtained from Invitrogen (Waltham, MA, USA). Antibodies against GAPDH (1:500) (sc-32233) were purchased from Santa Cruz Technology (Dallas, TX, USA). Donkey anti-rabbit IgG Alexa flour 555 (1:1000) (ab150074) was purchased from Abcam (Cambridge, UK). Insulin from bovine pancreas (16634), dexamethasone (D4902), 3-iobutyl-1-methylxanthine (IBMX) (I7018), rosiglitazone (R2408), toluidine blue (89640), fuchsin (47860), and Oil Red O were obtained from Sigma Aldrich (St. Louis, MO, USA).

### 2.2. DOE Preparation

*D. odorifera* wood was purchased from a Korean medicinal herb store in Seoul, South Korea. The plant was thoroughly washed, dried, and finely ground using a grinder. Powdered *D. odorifera* (100 g) was extracted with 70% ethanol at room temperature (RT) for 72 h. After evaporation of the solvent under vacuum, the extract (10 g) was reconstituted in 1% dimethyl sulfoxide (DMSO) to a concentration of 10 mg/mL (DOE) and then stored at −20 °C until use.

### 2.3. Cell Culture

Murine CT26 cells were cultured in an RPMI medium (Hyclone, Logan, UT, USA) supplemented with 10% fetal bovine serum (FBS, Hyclone) and 1% Penstrep (PS, Hyclone). Then, 3T3L1 preadipocytes and C2C12 myoblasts were cultured in DMEM (Hyclone) supplemented with 10% FBS and 1% PS. To induce C2C12 myoblast differentiation, when the cells reached 80% confluency, the old medium was discarded, and the cells continued to grow in the differentiation medium containing DMEM with 5% FBS and 1% PS for 6 days; the differentiation medium was changed every 2 days. The differentiation of 3T3L1 was induced with the DMEM medium containing 10% FBS, 1% PS, 0.5 mM IBMX, 1 μM dexamethasone, 2 μM rosiglitazone, and 10 μg/mL insulin for 3 days before switching to DMEM with 10% FBS, 1% PS, and 10 μg/mL insulin for 3 more days.

### 2.4. CT26 Conditioned Medium Preparation

When the CT26 cell density reached 90% confluency, the old medium was changed, and serum-free DMEM was added to each cell culture dish. After 48 h of incubation, CT26 cell supernatants were collected and centrifuged at 1500 rpm for 10 min to remove the cell debris. Centrifuged media were filtered through a 0.2 µm syringe filter and stored at −80 °C for further usage. To prepare the conditioned medium for the treatment, the filtered supernatant was diluted with complete DMEM (for 3T3L1) and with DMEM supplemented with 5% FBS (for C2C12) at a ratio of 80:20.

### 2.5. Cell Viability Assay

The cytotoxicity of DOE to C2C12 myoblasts and 3T3L1 preadipocytes was measured using the CCK-8 kit (Dongin, Seoul, Republic of Korea). In general, cells were seeded into 96-well plates at a density of 10000 cells/well in the growth medium overnight to allow for cells to attach to plates. Cells were incubated with different concentrations of DOE (0, 5, 10, 15, 20, 40, 80, 160, and 320 µg/mL) for 24 h. Then, 10 μL of CCK-8 was added to each well and incubated at 37 ℃ and 5% CO_2_ in humidity for 60 min. The absorbance at 450 nm was measured using an Epoch™ Microplate Spectrophotometer (Biotek, Emeryville, CA, USA). The results were averaged from five duplicate wells in each group.

### 2.6. Immunocytochemistry (ICC)

ICC was conducted to visualize the cell morphology of C2C12 myotubes in 8-well slides (PEZGS0816, Sigma, Darmstadt, Germany). After treatment with 80% CT26 CM and DOE for 24 h, cells were washed gently in PBS and fixed with ice-cold methanol for 20 min at RT. Permeabilization was performed with the Intracellular Staining Perm Wash Buffer (BioLegend, San Diego, CA, USA) for 15 min, followed by blocking in PBST containing 1% bovine serum albumin and glycine for 60 min at RT. The expression of MYH2 was detected using an anti-MYH2 antibody (1:400 dilution in perm 1X) with overnight incubation at 4 °C. After washing, cells were treated with donkey anti-rabbit IgG Alexa flour 555 for 2 h at RT in the dark. To stain the nuclei, the Vectashield Antifademounting medium with DAPI (LSbio, Shirley, MA, USA) was added to the cells, and visualization was conducted using a Zeiss LSM 900 confocal microscope (Zeiss, Oberkochen, Germany).

### 2.7. LADD Staining

LADD staining was performed as described previously [21]. Briefly, after C2C12 myotubes were treated in CT26 CM and DOE for 24 h, the old medium was removed, and cells were washed with PBS gently and fixed in EtOH 70% at room temperature for 15 min. Cells were then washed again with DPBS and LADD stain containing toluidine blue, and fuchsin was added to fully cover them for 5 min. Once the LADD stain was removed, the cells were repeatedly washed with distilled water until LADD stains stopped leaking into the water. Cell morphology was visualized under a microscope (KCS3-63S, Optinity, Gyeongi-do, Korea) equipped with Optiview, and the myotube diameter was measured using ImageJ (ImaheJ ij154).

### 2.8. Oil Red O Staining

After 3T3-L1 adipocytes were treated in CT26 CM and DOE for 24 h, the old medium was removed. Cells were washed with PBS and fixed in 4% paraformaldehyde at RT for 30 min. After washing with PBS, cells were stained with the working solution of Oil Red O for 30 min at RT and washed with water until the stain stopped leaking. Cell images were captured under a microscope. To quantify lipid accumulation, Oil Red O was extracted from cells using isopropanol, and the absorbance level was measured at a wavelength of 510 nm.

### 2.9. Glycerol Assay

A glycerol assay was used to assess the lipolysis level. Mouse serum and cell culture supernatants obtained after treatment were collected, and the released glycerol was measured using a glycerol assay kit (Sigma Aldrich, Saint Louis, MO, USA).

### 2.10. Animal Study

**Figure 1:**
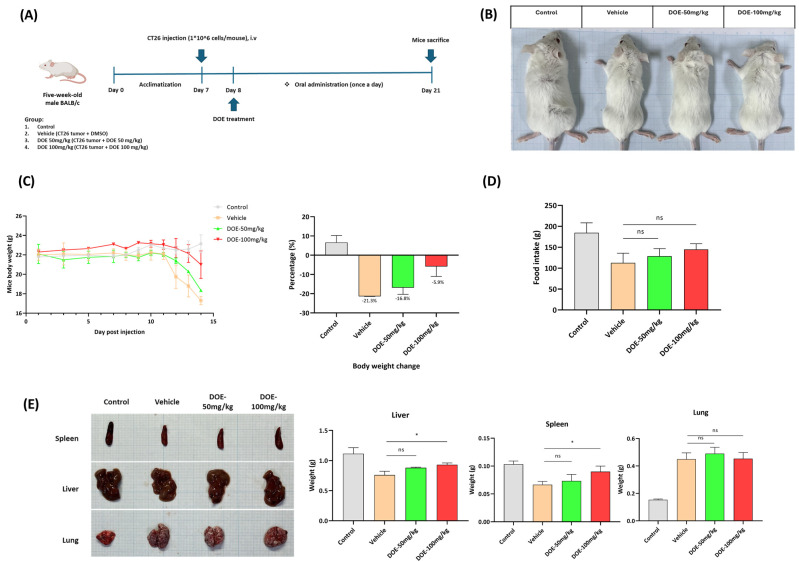
*Dalbergia odorifera* plant extract improved the symptoms of cancer cachexia in CT26-bearing mice. A schematic representation of the experimental design is illustrated. In general, CT26 tumor cells were intravenously injected into 6-week-old Balb/c mice on day 7, and DOE (50, 100 mg/kg) was orally administrated for 14 days starting after 1 day of tumor inoculation. Healthy mice and the vehicle group received an equal volume of solvent. Body weight and food intake were recorded every day (**A**). Representative pictures of mice from the four groups at the end of the experiment (**B**) and the body weight curve show changes in body weight in the four treatment groups over the experiment (**C**). Mouse body weight (**D**), food consumption (**E**), and different organs were compared among the four groups. Values are expressed as the mean ± SEM. A one-way ANOVA was performed with * *p* < 0.05, ns: not significant.

To investigate the effects of DOE in ameliorating the symptoms of cancer cachexia in vivo, a murine model of CT26 cancer cachexia was established considering the fact that the CT26 tumor-bearing mouse model is one of the most well-accepted systems for studying the etiopathogenesis and drug screening related to cancer cachexia. Balb/c male mice aged 5 weeks were purchased from DBL, Korea. The animals were maintained in conditions of constant temperature and humidity with 12 h light/dark cycles. For acclimation to the new environment, they were allowed to drink and eat freely for 1 week. On day 7, mice were injected with CT26 cells (10^6^ cells/200 µL) intravenously to induce cancer cachexia, except for those in the control group that received DPBS. The next day, mice were randomly divided into four groups: the control group (healthy mice orally administered 1% DMSO), the vehicle group (CT26-bearing mice receiving 1% DMSO by gavage), and two DOE-treated groups (CT26-bearing mice orally administered DOE at concentrations of 50 mg/kg BW and 100 mg/kg BW). The treatment continued for 14 days (Figure 1A). At this time, the body weight and food intake of the mice were measured daily. After 14 days, treatment was stopped, and mice were euthanized with 70 mg/kg IP alfaxalone and 10 mg/kg IP xylazine. The lung, spleen, liver, gastrocnemius, and epididymal fat were removed and weighed. Mouse blood samples were collected in an e-tube, left undisturbed at RT for 30 min to allow for the blood to clot, and then centrifuged (2000× *g*, 10 min, 4 °C) to obtain serum.

### 2.11. Hematoxylin and Eosin (H&E) Staining

The gastrocnemius tissues and epididymal fats were fixed in 10% neutral buffer formalin for 24 h and embedded in paraffin after dehydration. These samples were sliced into 5 µm sections, mounted on glass slides dewaxed with xylene, rehydrated in a graded ethanol series to water, and finally stained with H&E. Five images were taken randomly for each sample using a microscope (KB-600, Optinity) equipped with Optiview. The average cross-sectional area (CSA) of the adipocytes and minimal Feret’s diameter of muscle fibers were assessed using Image J.

### 2.12. Western Blot Analysis

C2C12 myotubes, 3T3L1 adipocytes, gastrocnemius, and epididymal fat tissues were lysed with a PRO-PREP solution (iNtRON BIOTECHNOLOGY, Gyeonggi, Republic of Korea), in accordance with the manufacturer’s instructions. In total, 30 μg of protein was separated on a 12% polyacrylamide gel and transferred to polyvinylidene difluoride membrane (Millipore Corporation, Bedford, MA, USA). Membranes were blocked in 5% skim milk at RT for 1 h and then incubated with primary antibodies at 4 °C overnight. The blots were washed in Tris-buffered saline containing 1% Tween^®^ 20 (TBST) five times every 10 min, and probed with the corresponding horseradish peroxidase-conjugated secondary antibodies for 1 h at RT. After washing the membranes again with TBST, West-Q Pico Dura ECL Solution (GenDEPOT, Katy, TX, USA) was added to visualize the protein bands, and the chemiluminescent signals were detected by Invitrogen iBright 1500 (Waltham, MA, USA). GAPDH was used as the internal control.

### 2.13. Reverse-Transcription Quantitative Polymerase Chain Reaction (qPCR)

Total RNA was isolated from cells and tissues using TRI Reagent (MRC, Columbus, OH, USA), following the manufacturer’s instructions. RNA concentration was measured using an Epoch™ Microplate Spectrophotometer (Biotek, CA, USA) and diluted in nuclease-free water (NFW) to a final concentration of 50 ng/μL. For each sample, qPCR was performed with a one-step AccuPower^®^ GreenStar™ RT-qPCR Master Mix (Bioneer, Daejeon, Republic of Korea) in triplicate. Each qPCR reaction contained 2 µL of the RNA template, 10 pmole of each gene-specific primer, 10 µL of the master mix, and 6 µL of NFW. Temperature control and fluorescence measurement were conducted using a Rotor Gene Q thermocycler (QIAGEN, Hilden, Germany). GAPDH was used for internal control data normalization. The PCR conditions were set as follows: 50 °C for 15 min for reverse transcription, initial denaturation step at 95 °C for 5 min, followed by 45 cycles of denaturation at 95 °C for 10 s, annealing at 60 °C for 15 s, and elongation at 72 °C for 20 s. Relative target gene expression was calculated using the 2^−ΔΔCt^ method. The primer sets for qRT-pCR are described in Table 1.

### 2.14. Statistical Analysis

Data were processed with GraphPad Prism software 8 (GraphPad Software Inc., San Diego, CA, USA). The results are presented as the mean ± SEM and were compared using a one-way analysis of variance. Differences were considered significant at * *p* < 0.05, ** *p* < 0.01, *** *p* < 0.001, or **** *p* < 0.0001.

## 3. Results

### 3.1. DOE Improved the Loss of Body Weight, Food Intake, and Releasing of Glycerol in the Serum Samples of CT26-Bearing Mice

To investigate the anti-cachexia effects of DOE, CT26-bearing mice were orally administered 50 and 100 mg/kg of DOE continuously for 14 days. Mouse body weight was measured every day, and CT26 bearers showed significant loss from day 10 after tumor inoculation (Figure 1B,C). DOE treatment improved the symptoms of cancer cachexia in a dose-dependent manner. At the time of sacrifice, the control group increased approximately 7% in their body weight. CT26-bearing mice showed an approximately 21% body weight loss compared with the initial body weight; meanwhile, in the DOE-50 mg/kg-treated group, the mouse body weight loss was approximately 17%. In the DOE-100 mg/kg-treated group, the loss was only 6% (Figure 1C). Food intake was compared among the four groups and indicated that DOE treatment can improve mice’s appetite but was not significant (Figure 1D). The sign of wasting was also observed in the spleen and liver samples of the diluent--only-treated group, but somewhat recovered when mice were administered DOE; meanwhile, no difference in the size of the tumor located in the lung of three CT26-bearing groups was found (Figure 1E).

### 3.2. DOE Attenuates Skeletal Muscle Atrophy in CT26-Bearing Mice

**Figure 2:**
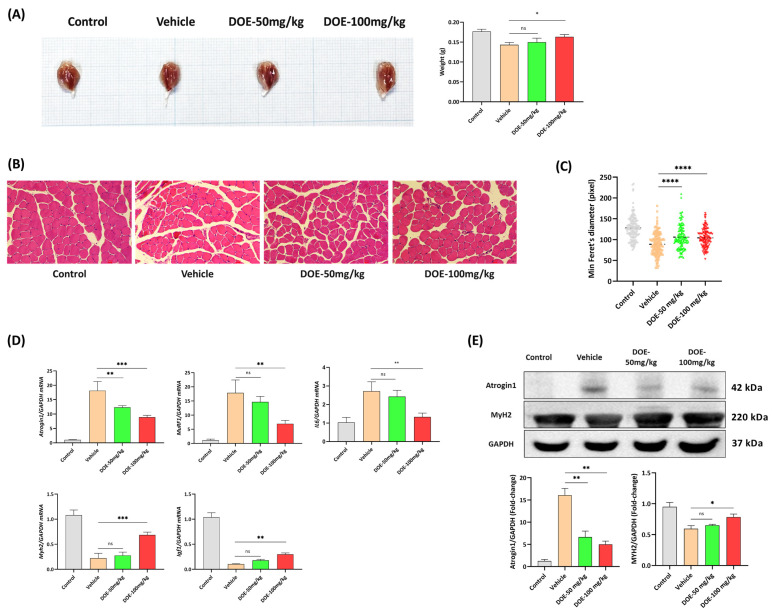
DOE ameliorates skeletal muscle wasting in CT26 tumor-bearing mice. Representative images and weight of the gastrocnemius muscle collected from mice in the four groups (**A**). The hematoxylin and eosin (H&E) staining of gastrocnemius sections from the four groups with scale bars = 100 px (**B**). The minimal Feret’s diameter of 100 muscle fibers from each group was measured using ImageJ (**C**). The mRNA expressions of Atrogin1, MuRF, IL6, Myh2, and Igf1 in the skeletal muscles were assessed by qRT-PCR (*n* = 6). mRNA was normalized against GAPDH. * *p* < 0.05, ** *p* < 0.01, *** *p* < 0.001, **** *p* < 0.0001, ns: not significant versus the vehicle control (**D**). Western blot analysis of MuRF1, Atrogin-1, MYH2, FOXO3a, *p*-FOX3a, AKT, and *p*-AKT expression levels in the gastrocnemius muscles of different groups. GAPDH was used as the internal control (**E**).

Muscle atrophy is one of the main characteristics of cancer cachexia. Here, the ability of DOE in preventing muscle wasting in CT26-bearing mice was tested. Significant loss of gastrocnemius muscle was observed in tumor-bearing mice; however, DOE treatment could prevent this change at the dose of 100 mg/kg (Figure 2A).

Histopathological analysis of gastrocnemius muscles from the four different groups performed by H&E staining revealed that the muscle fibers of the CT26-bearing mice administered with diluent only were shrunken compared with the control group but were markedly improved with DOE treatment (Figure 2B). By measuring the CSA of muscle fibers, CT26-bearing mice had many smaller fibers, suggesting that the whole muscle undergoes atrophy in the presence of CT26 tumor, and a shift was found toward bigger fibers in the DOE-treated groups (Figure 2C). The reduction in muscle fiber size was consistent and proportional to skeletal muscle weight loss.

The mRNA expressions of Atrogin1 and MuRF1 genes were elevated more than 15-fold in cachectic mice compared with the control group. After DOE intervention, the expressions of the Atrogin1 and MuRF1 mRNA expression were significantly downregulated, particularly in the 100 mg/kg DOE-treated group. Moreover, Myh2 and Igf1 expression levels were dose-dependently increased in response to DOE treatment. Furthermore, the expression of IL-6, a proinflammatory cytokine, was reduced two-fold after treatment with 100 mg/kg DOE, indicating the function of DOE in reducing inflammation in muscle samples (Figure 2D). Consistent with the mRNA expression data, the protein expression levels of Atrogin1 were upregulated in CT26 bearers, whereas MYH2 levels slightly decreased. However, DOE treatment at two concentrations could reverse the effect of muscle atrophy induced by a CT26 tumor through a reduced expression level of Atrogin1 and an increased expression level of MYH2 compared with CT26-bearing mice, which received diluent only (Figure 2E).

### 3.3. DOE Prevents the Loss of Adipose Tissue in CT26-Bearing Mice

**Figure 3:**
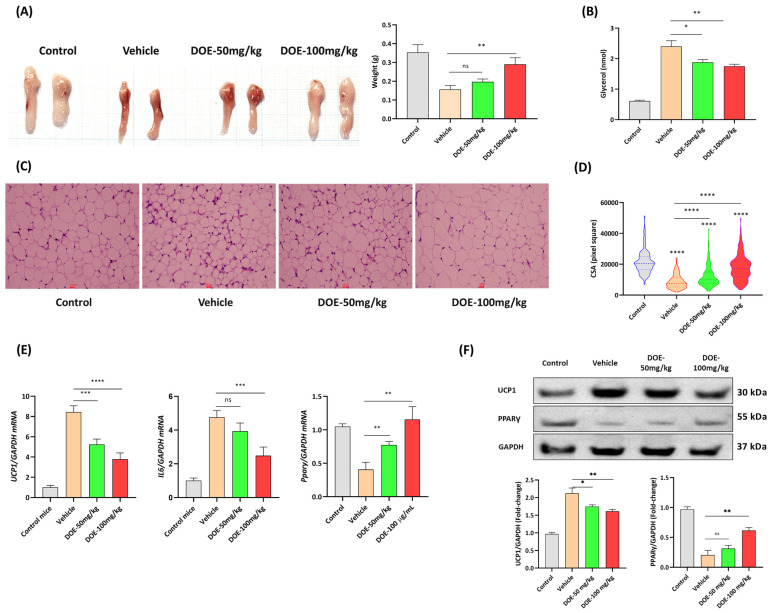
DOE attenuates adipose tissue loss in CT26 tumor-bearing mice. Representative images and weight of epididymal fats collected from mice in the four groups (**A**). Glycerol was measured to evaluate the degree of lipolysis in the serum samples (**B**). The hematoxylin and eosin (H&E) staining of epididymal sections from the four groups with scale bars = 100 px (**C**). The cross-sectional area (CSA) of 100 adipocytes from each group was measured using ImageJ (**D**). mRNA expression levels of Ucp1, IL6, and Ppary were evaluated by RT-qPCR. mRNA was normalized against GAPDH (*n* = 6). * *p* < 0.05, ** *p* < 0.01, *** *p* < 0.001, **** *p* < 0.0001, ns: not significant versus the vehicle control (**E**). Protein expressions of UCP1, PPARy, AKT, and *p*-AKT in epididymal fats were evaluated using Western blot, and GAPDH was used as the internal control (**F**).

The level of adipose loss was also assessed and compared among the four groups. A significant decrease in epididymal fat was observed in the CT26-bearing group compared with the healthy group; however, DOE treatment at the dose of 100 mg/kg could remarkably attenuate this loss (Figure 3A).

Glycerol in mouse serum samples was also measured to evaluate the degree of lipolysis. Its concentration was the highest in CT26-bearing mice and gradually reduced in the DOE-treated group (Figure 3B).

The morphology of adipose cells was monitored by H&E staining and indicated that DOE supplementation could inhibit the reduction in adipocyte size (Figure 3C). The average CSA and frequency distribution indicated that the CSA of adipocytes was the lowest in CT26-bearing mice administered diluent only, and that DOE treatment could recover the cell size significantly (Figure 3D). RT-qPCR was conducted to measure the mRNA expression levels of lipolysis-related and adipogenesis-related genes. The upregulation of lipolysis markers Ucp1 and IL6 in the epididymal fats of CT26 bearers were improved by a two-fold decrease in the 100 mg/kg-treated group compared with the control group. In addition, the mRNA expression level of Pparγ was downregulated in the CT26-bearing group but was significantly enhanced by DOE (Figure 3E). Similar to the RT-qPCR results, Western blot of UCP1 showed an increase in the protein expression levels in the epididymal fat samples of CT26-bearing mice, whereas DOE treatment neutralized this effect. At the same time, the PPARγ protein level exhibited an increase in response to 100 mg/kg DOE treatment compared to CT26-bearing mice (Figure 3F).

### 3.4. DOE Prevents Muscle Atrophy (In C2C12 Myotubes) Induced by Cancer Cachexia

**Figure 4:**
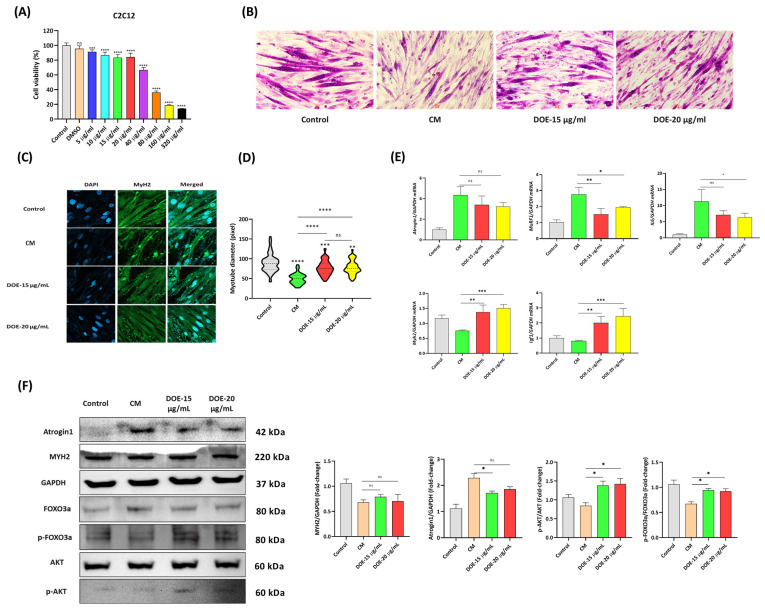
DOE prevents the CM-induced atrophy of C2C12 myotubes. The toxicity of DOE to C2C12 myoblasts was checked by CCK-8 (**A**). Cell morphology of myotubes from different groups was visualized by LADD (20X magnification) (**B**) or ICC when nuclei were detected by DAPI (blue) and MYH2 by Alexa Flour 555 at 40X magnification (**C**). Myotube diameter was measured using ImageJ (**D**). qRT-PCR was conducted to evaluate the mRNA expression levels of Atrogin1, MuRF, IL6, Myh2, and Igf1 after 24 h of treatment with CM and DOE (**E**), and the protein expression levels of MuRF1, Atrogin1, MYH2, FOXO3a, *p*-FOXO3a, AKT, and *p*-AKT were evaluated using Western blot (**F**). A one-way ANOVA was carried out to determine significant differences (* *p* < 0.05, ** *p* < 0.01, *** *p* < 0.001, **** *p* < 0.0001, ns: not significant).

Then, whether DOE could attenuate the atrophy of myotubes induced by CT26 CM was investigated. The cytotoxicity of DOE to C2C12 myoblasts was measured using CCK-8, which indicated that DOE markedly decreased cell viability at concentrations of ≥40 µg/mL after 24 h. Therefore, 15 and 20 µg/mL were chosen for the subsequent experiments (Figure 4A). Cell morphology was visualized by ICC using MYH2 antibody, and LADD staining indicated that the myotube diameter was significantly reduced when C2C12 myotubes were treated with CM. Meanwhile, the addition of DOE at two concentrations remarkably restored the cell size (Figure 4B,C). The diameter of the myotubes was measured using ImageJ, and the results indicated that DOE treatment could enhance the diameter compared with CM treatment. However, no significant difference was found between the two chosen concentrations (Figure 4D). As shown in Figure 4E, the mRNA expression levels of Atrogin1, MuRF, and IL6 were increased, whereas those of MyH2 and Igf1 were downregulated considerably in response to CM treatment. However, DOE treatment could reverse this effect. Western blot analysis to measure protein expression levels also showed comparable results. Alongside that, DOE treatment significantly increased the protein levels of *p*-AKT and *p*-FOXO3a in CM-treated C2C12 cells, suggesting the involvement of DOE in regulating the AKT signaling pathway (Figure 4F). Overall, our findings revealed that DOE inhibited CM-induced atrophy in C2C12 cells.

### 3.5. DOE Prevents Lipolysis (In 3T3L1 Adipocytes) Induced by Cancer Cachexia

**Figure 5:**
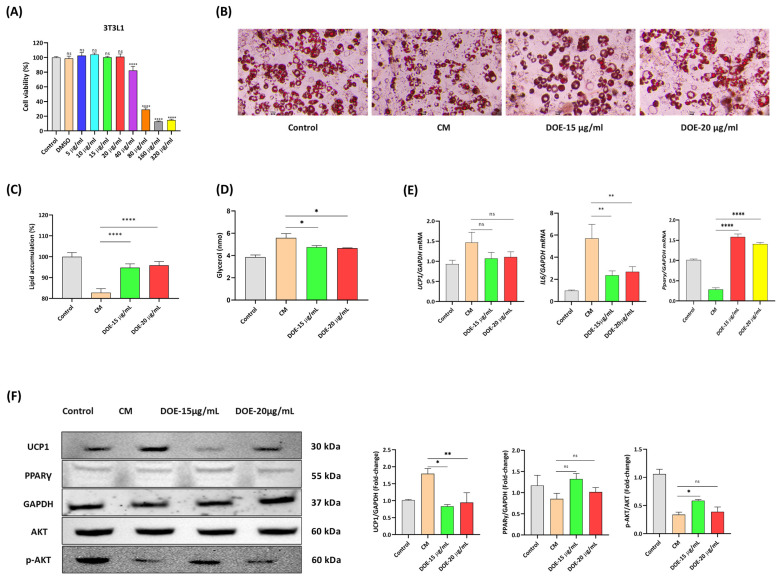
DOE prevents CM-induced lipolysis in 3T3L1 adipocytes. The cytotoxicity assay of the DOE to 3T3L1 preadipocytes was assessed using CCK-8 (**A**). Cell morphology was visualized by Oil Red O staining (**B**), and lipid accumulation was compared among the four groups (**C**). Glycerol was measured to evaluate the degree of lipolysis (**D**), and the mRNA expression levels of Ucp1, IL6, and Ppary were evaluated for all the groups (**E**). Protein levels of UCP1, PPARγ, AKT and *p*-AKT via Western blot (**F**). Data are presented as the mean ± SEM. Significance was determined by a one-way ANOVA (* *p* < 0.05, ** *p* < 0.01, **** *p* < 0.0001, ns: not significant).

The efficacy of DOE in preventing lipolysis in 3T3L1 adipocytes was investigated. Similar to the results of C2C12 myoblasts, with 40 µg/mL, DOE showed toxicity to 3T3L1 after 24 h. Thus, 15 and 20 µg/mL were chosen for further experiments (Figure 5A). Cell morphology was visualized by Oil Red O staining (Figure 5B). The accumulation of lipid droplets of 3T3L1 adipocytes was compared among the four groups, and a clear difference was found, that is, the highest in the control and the lowest in the CT26-treated group, indicating that DOE treatment can somewhat prohibit the loss of lipid droplets (Figure 5C). Furthermore, glycerol concentration was the highest in the CM-treated group, and DOE treatment helped reduce the release of glycerol from adipocytes induced by CT26 CM (Figure 5D).

The CT26 CM-induced expression of Ucp1 and IL6 mRNA levels was drastically inhibited by DOE. Moreover, the effect of CM on downregulating marker genes of lipogenesis such as Pparγ in 3T3L1 adipocytes was abolished in the DOE-treated groups (Figure 5E). At the protein level, CT26 CM increased UCP1 and decreased PPARγ expression significantly; however, these effects were neutralized by DOE. *p*-AKT was also upregulated in 3T3L1 cells treated with DOE (Figure 5F). Here, 15 µg/mL DOE showed better results than 20 µg/mL; thus, for our subsequent experiments, the treatment dose will be 15 µg/mL. Taken together, our results indicated that DOE might have a protective effect against CM-induced lipolysis in 3T3L1 cells.

### 3.6. DOE Improves Muscle Wasting and Lipolysis by Promoting the PI3K/AKT Signaling Pathway

DOE treatment significantly increased the protein levels of *p*-AKT in CT26 CM-treated C2C12 and 3T3L1 cells. To confirm the involvement of DOE in regulating the AKT signaling pathway via PI3K, we used wortmannin, a PI3K inhibitor. C2C12 myotubes and 3T3L1 adipocytes were pretreated with wortmannin for 1 h before the addition of CT26 CM and 15 µg/mL of DOE.

**Figure 6:**
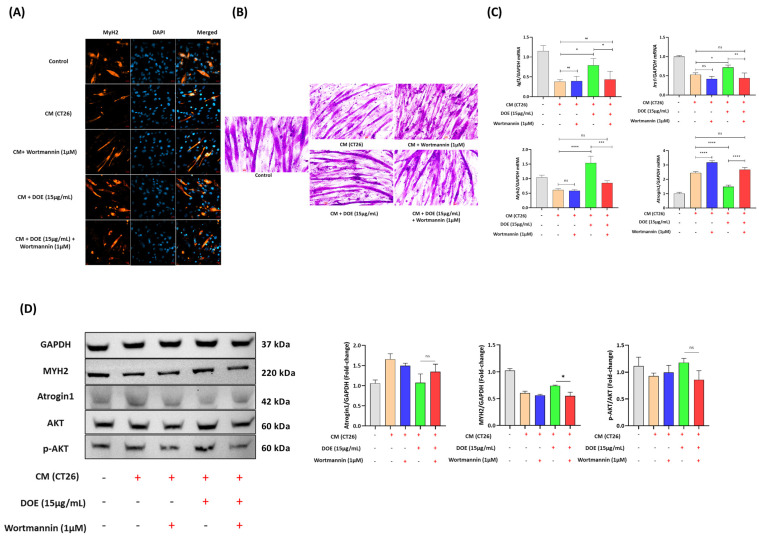
PI3K inhibitor (wortmannin) inhibited the therapeutic effects of DOE in preventing muscle wasting. Wortmannin was added to inhibit the AKT signaling pathway. In general, 1 µM wortmannin was pretreated to cells 1 h before the addition of CM and DOE. After 24 h, cells were stained and harvested for further analysis. Representative images of myofibers were immunostained with the antibody for MYH2 (orange) and counter-stained with DAPI (blue) for visualization of the nucleus (20× magnification) (scale bar = 20 μm) (**A**), and C2C12 myotubes by LADD staining (20× magnification) (scale bar = 100 px) (**B**). The expression levels of Igf1, Irs1, Myh2, and Atrogin-1 mRNA in myotubes treated with 15 ug/mL DOE with or without wortmannin for 24 h were measured, and mRNA was normalized against GAPDH (**C**). The protein levels of AKT, *p*-AKT, Atrogin1, and MYH2 were measured via Western blot analysis (**D**). Data are presented as the mean ± SEM. The statistical significance was determined by a one-way ANOVA (* *p* < 0.05, ** *p* < 0.01, *** *p* < 0.001, or **** *p* < 0.0001, ns: not significant).

For C2C12 cells, ICC and LADD staining results demonstrated that the administration of DOE and wortmannin led to a reduction in cell diameter in comparison with DOE treatment alone (Figure 6A,B). In addition, the mRNA expression and protein levels of Atrogin1 could not be suppressed, and Myh2 levels could not recover in the combined treatment group (Figure 6C,D). The expression levels of Igf1 and Irs1, which are the upstream effectors of PI3K/AKT, were evaluated at the mRNA level, which showed that the efficacy of DOE in enhancing Igf1 and Irs1 expression was abolished through the addition of wortmannin (Figure 6C). Moreover, *p*-AKT, which was activated by DOE treatment, was downregulated when cells were co-treated with wortmannin (Figure 6D).

**Figure 7:**
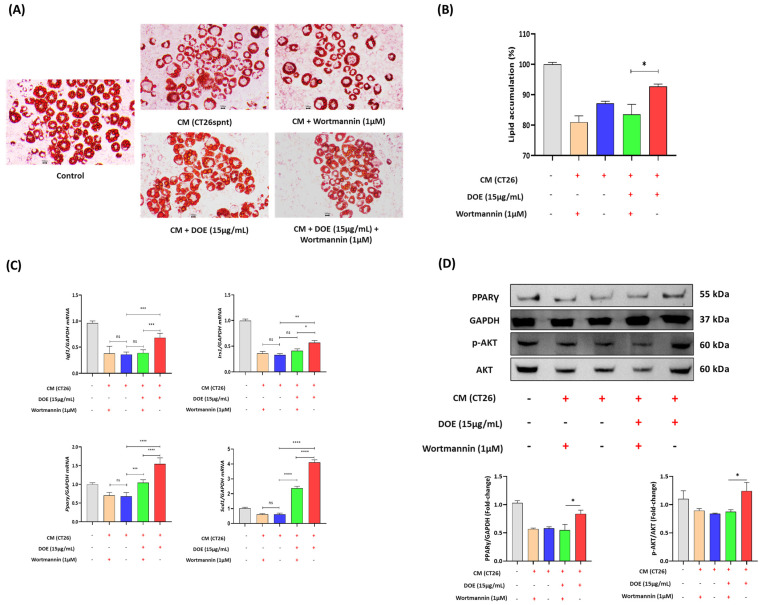
PI3K inhibitor (wortmannin) inhibited the therapeutic effects of DOE in promoting lipogenesis. Wortmannin was added to inhibit the AKT signaling pathway. In general, 1 µM wortmannin was pretreated to cells 1 h before the addition of CM and DOE. After 24 h, cells were stained and harvested for further analysis. Oil Red O staining (**A**) and lipid accumulation measurement (**B**) of 3T3L1 adipocytes after CM, DOE, and wortmannin treatments in different groups were conducted. The mRNA levels of Igf1, Irs1, Ppary, and Scd1 were evaluated using qPCR, and the results are expressed relative to GAPDH (**C**). Western blot analysis of the protein expression levels of AKT, *p*-AKT, and PPARγ in the 3T3-L1 mature adipocytes of different groups (**D**). Data are presented as the mean ± SEM. The statistical significance was determined by a one-way ANOVA (* *p* < 0.05, ** *p* < 0.01, *** *p* < 0.001 or **** *p* < 0.0001, ns: not significant).

In 3T3L1 adipocytes, wortmannin pretreatment followed by the addition of CT26 CM and DOE reduced the accumulation of lipid droplets significantly (Figure 7A,B). The mRNA expression levels of Igf1, Irs1, Pparγ, and Scd1, which were increased by DOE treatment, were suppressed in the presence of wortmannin (Figure 7C). The CM-induced downregulation of *p*-AKT and PPARγ was reversed by DOE treatment. However, this effect was diminished by the addition of 1 µM wortmannin (Figure 7D).

These results indicated that the protective effects of DOE in muscle wasting and lipolysis were attenuated by the inhibition of the PI3K/AKT signaling pathway.

## 4. Discussion

Cancer cachexia is a multifactorial syndrome that is manifested by the loss of body weight, and muscle and adipose wasting [22]. Despite the huge effects on patients with cancer, currently, no standard treatments or interventions are available for patients with this condition [23]. Anamorelin, a ghrelin receptor agonist, has been applied in the clinical treatment of patients with cancer cachexia in Japan to improve appetite and body weight loss [24,25]. Despite the increase in lean body mass, no improvement in physical function was found among patients with cancer cachexia, making it impossible to be approved by EMA for clinical therapy [26,27]. Thus, discovering new cancer cachexia treatment is necessary.

In recent years, accumulating evidence suggests that medicinal plants and their phytochemicals hold great potential as an alternative and promising treatment strategy for alleviating cancer cachexia symptoms owing to their high efficacy and minimal toxicity [4,28]. D. odorifera has been used for a long time as a traditional herbal medicine in China and Korea to promote blood circulation, relieve pain, and eliminate blood stasis [20]. Certain compounds identified from DOE, including flavonoids, quinines, and phenolic constituents, exhibit diverse biological activities such as anti-inflammation and antitumor activity. In addition, formononetin, which is a flavonoid, was demonstrated to exert an anti-muscle atrophy effect and promote satellite cell function via the PI3K/AKT/FoxO3a pathway [29].

In this study, oral DOE therapy demonstrated potential as a new anticancer cachexia candidate by increasing body weight, skeletal muscle mass, epididymal fat, and food intake in a dose-dependent manner. By intravenous injection, the tumor cells formed nodules in the lungs, and no differences were observed in the lung samples among the three CT26-bearing groups, suggesting that the anti-cachexia effect of DOE appeared to be unrelated to its antitumor effect.

Skeletal muscles are one of the major compartments of the human body, which are essential for various biological processes, including movement and respiration [8]. Muscle mass loss occurs through atrophy, which is characterized by increases in protein degradation processes, particularly the ATP-dependent ubiquitin–proteasome proteolytic pathway. This process is commonly seen in cancer cachexia, accompanied by the upregulation of two key muscle-specific ubiquitin ligases, namely, Atrogin1 and MuRF1 [8,30]. In addition, during cachexia, both patients with cancer and mouse models show reduced circulating levels of the anabolic factor insulin-like growth factor-1 (IGF-1), alongside the development of insulin resistance [8]. Among the signaling pathways that regulate muscle development, the AKT signaling pathway plays a central role in both catabolism and anabolism [22,30]. In general, the binding of IGF-1 to its receptor triggers the activation of several intracellular kinases, including IRS1, which will eventually catalyze PI3K and AKT phosphorylation [9]. Once AKT is phosphorylated, the phosphorylation of FOXO3a, a protein in the Forkhead family of transcription factors, occurs. This phosphorylation prevents FOXO3a from translocating into the nucleus, thereby inhibiting its transcriptional functions and leading to the suppression of Atrogin1 and MuRF1 [22].

In this study, the Igf1 mRNA expression level was upregulated in response to DOE administration, which eventually resulted in MuRF1 and Atrogin1 reduction. Moreover, the protein expression levels of *p*-AKT and *p*-FOXO3 were increased in these treated groups, indicating the relevance of DOE in regulating this signaling pathway. AKT phosphorylation will also lead to mTOR activation, therefore promoting protein synthesis [15,31]. According to the qRT-PCR and Western blot results, DOE treatment significantly increased the expression level of MyH2—a major structural protein of myotubes—suggesting that DOE promotes muscle protein synthesis and prevents degradation.

In addition to the muscles, adipose tissue is susceptible to the effects of cancer cachexia [22,32]. Adipose tissue is an important organ that stores energy for the body, which is classified as white adipose tissue (WAT) and brown adipose tissue with distinct functions. To maintain proper function, adipose tissue also depends on the AKT signaling pathway [33]. AKT activation will drive mTORC1, which enhances the expression level of PPARγ [31]. As a transcription factor, PPARγ regulates the expression of lipogenic genes such as Scd1, and modulates the lipid synthesis inside adipocytes [34,35]. This pathway, under the development of cancer cachexia, is affected. Owing to mediators released by tumors or host cells, WAT undergoes a browning process, resulting in increased lipid mobilization and energy expenditure [36]. During this process, the expression level of UCP1, an integral membrane protein unique to brown adipocyte mitochondria, increases [36]. In addition, IL-6, which is a critical inflammation mediator of cancer cachexia driving the expression level of UCP1, is upregulated [36]. In this study, the expression levels of UCP1 and IL6 upon cancer cachexia were increased in both adipose tissue and adipocytes; however, this effect was reversed by DOE treatment. Pparγ and Scd1 mRNA expression levels, which were prohibited by cancer cachexia, were recovered by DOE administration. *p*-AKT phosphorylation was also activated in the DOE-treated sample, indicating the efficacy of DOE in preventing adipose wasting via the AKT signaling pathway.

**Figure 8:**
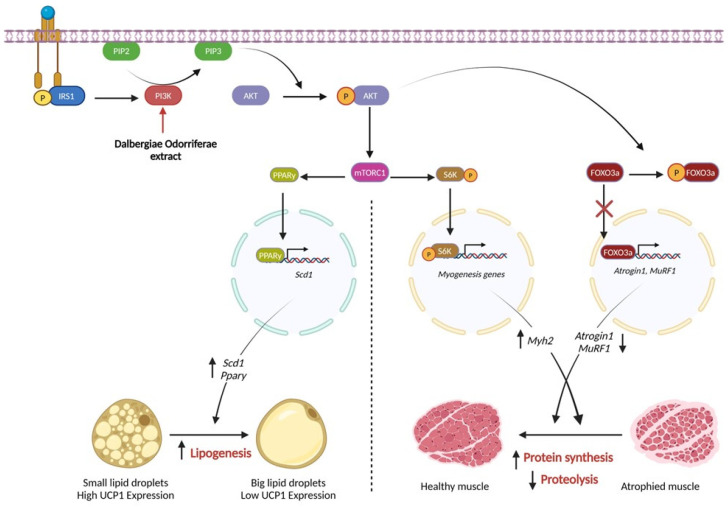
DOE is involved in the Akt signaling pathway to regulate protein synthesis and lipogenesis. Schematic illustration of the mechanism. A diagram of the proposed mechanism shows that DOE attenuates cachexia symptoms by targeting AKT via PI3K. DOE promotes the phosphorylation of PI3K and then AKT, leading to the downregulation of Atrogin1 and MuRF1 expression levels, and increasing mTOR activation, thus increasing protein synthesis and lipogenesis.

In this study, wortmannin, a specific inhibitor of PI3K, was used to determine whether DOE activated AKT through the PI3K/AKT pathway or through other alternative pathways [37]. Wortmannin treatment attenuated AKT activation induced by DOE, confirming that the efficacy of DOE is dependent on the PI3K pathway. The proposed mechanism of this plant extract is illustrated in Figure 8.

Cancer cachexia is associated with systemic inflammation, and some proinflammatory cytokines were found to induce muscle atrophy and lipolysis in cachexia, including IL-6 and TNF-α [38]. In this study, we analyzed skeletal muscle, epididymal fat, C2C12 myotubes, and 3T3L1 adipocyte mRNA levels, and found that DOE treatment affects the expression of IL-6, suggesting that DOE may attenuate cancer cachexia by affecting the production of proinflammatory cytokines by immune cells and tumor cells. This possibility needs to be further evaluated.

In summary, this study provides compelling evidence that DOE alleviates cancer cachexia-induced muscle atrophy and lipolysis in both cells and cachectic CT26-bearing mice through the activation of the PI3K/AKT signaling pathway. These findings contribute to our understanding of the molecular mechanisms of cachexia and highlight the potential of DOE as a novel therapeutic approach. In the future, the focus will be on identifying active compounds in DOE and elucidating the mechanisms involved in the anti-cachectic effects. Additionally, further research and clinical validation are essential to fully realize the therapeutic potential of DOE in cancer cachexia.

## 5. Conclusions

Current treatment options for cancer cachexia remain limited due to the complex and poorly understood etiology of the disease. In this study, we identified that DOE, a plant extract commonly used in traditional medicine in Korea and China, demonstrated significant anti-cachexia effects. Specifically, DOE treatment in a mouse model mitigated the loss of body weight, skeletal muscle mass, and adipose tissue, while also reversing myotube atrophy and adipocyte lipolysis in vitro. These findings suggest a promising role for plant-derived therapies in combating cancer cachexia. However, further validation of these results in additional animal models is necessary before progressing to clinical translation in humans.

## Figures and Tables

**Table 1:** Primers for qRT-PCR.

Primer Name	Primer Sequence (5′-3′)
Atrogin1-F	ATGCACACTGGTGCAGAGAG
Atrogin1-R	TGTAAGCACACAGGCAGGTC
MuRF1-F	CACGAAGACGAGAAGATCAACATC
MuRF1-R	AGCCCCAAACACCTTGCA
IL6-F	CTTCTTGGGACTGATGCTGGTGAC
IL6-R	TCTGTTGGGAGTGGTATCCTCTGTG
Irs1-F	TGTCACCCAGTGGTAGTTGCTC
Irs1-R	CTCTCAACAGGAGGTTTGGCATG
Myh2-F	AGGCGGCTGAGGAGCACGTA
Myh2-R	GCGGCACAAGCAGCGTTGG
Igf-F	GGACCAGAGCCCTTTGCGG
Igf-R	GGCTGCTTTTGTAGGCTTCAGTGG
Gapdh-F	AACATCATCCCTGCTTCCACT
Gapdh-R	GGCAGGTCAGATCCACAAC
Ucp1-F	AAGCTGTGCGATGTCCATGT
Ucp1-R	AAGCCACAAACCCTTTGAAAA
Irs1-F	TGTCACCCAGTGGTAGTTGCTC
Irs1-R	CTCTCAACAGGAGGTTTGGCATG
Pparγ-F	CAAGAATACCAAAGTGCGATCAA
Pparγ-R	GAGCTGGGTCTTTTCAGAATAATAAG
Scd1-F	CTGACCTGAAAGCCGAGAAG
Scd1-R	GCGTTGAGCACCAGAGTGTA

## Data Availability

The original contributions presented in the study are included in the article, further inquiries can be directed to the corresponding authors.

## References

[1] Fearon K., Strasser F., Anker S.D., Bosaeus I., Bruera E., Fainsinger R.L., Jatoi A., Loprinzi C., MacDonald N., Mantovani G. (2011). Definition and classification of cancer cachexia: An international consensus. Lancet Oncol..

[2] Ferrer M., Anthony T.G., Ayres J.S., Biffi G., Brown J.C., Caan B.J., Feliciano E.M.C., Coll A.P., Dunne R.F., Goncalves M.D. (2023). Cachexia: A systemic consequence of progressive, unresolved disease. Cell.

[3] McGovern J., Dolan R.D., Skipworth R.J., Laird B.J., McMillan D.C. (2022). Cancer cachexia: A nutritional or a systemic inflammatory syndrome?. Br. J. Cancer.

[4] Kim A., Im M., Gu M.J., Ma J.Y. (2016). Citrus unshiu peel extract alleviates cancer-induced weight loss in mice bearing CT-26 adenocarcinoma. Sci. Rep..

[5] Schmidt S.F., Rohm M., Herzig S., Diaz M.B. (2018). Cancer Cachexia: More Than Skeletal Muscle Wasting. Trends Cancer.

[6] Kadakia K.C., Hamilton-Reeves J.M., Baracos V.E. (2023). Current Therapeutic Targets in Cancer Cachexia: A Pathophysiologic Approach. Am. Soc. Clin. Oncol. Educ. Book.

[7] Tisdale M.J. (2002). Cachexia in cancer patients. Nat. Rev. Cancer.

[8] Porporato P.E. (2016). Understanding cachexia as a cancer metabolism syndrome. Oncogenesis.

[9] Siddiqui J.A., Pothuraju R., Jain M., Batra S.K., Nasser M.W. (2020). Advances in cancer cachexia: Intersection between affected organs, mediators, and pharmacological interventions. Biochim. Biophys. Acta (BBA)—Rev. Cancer.

[10] Johns N., Stephens N.A., Fearon K.C. (2013). Muscle wasting in cancer. Int. J. Biochem. Cell Biol..

[11] Dahlman I., Mejhert N., Linder K., Agustsson T., Mutch D.M., Kulyte A., Isaksson B., Permert J., Petrovic N., Nedergaard J. (2010). Adipose tissue pathways involved in weight loss of cancer cachexia. Br. J. Cancer.

[12] Geremia A., Sartori R., Baraldo M., Nogara L., Balmaceda V., Dumitras G.A., Ciciliot S., Scalabrin M., Nolte H., Blaauw B. (2022). Activation of Akt-mTORC1 signalling reverts cancer-dependent muscle wasting. J. Cachexia Sarcopenia Muscle.

[13] Sartori R., Romanello V., Sandri M. (2021). Mechanisms of muscle atrophy and hypertrophy: Implications in health and disease. Nat. Commun..

[14] Yang W., Huang J., Wu H., Wang Y., Du Z., Ling Y., Wang W., Wu Q., Gao W. (2020). Molecular mechanisms of cancer cachexia-induced muscle atrophy (Review). Mol. Med. Rep..

[15] Chen L., Chen L., Wan L., Huo Y., Huang J., Li J., Lu J., Xin B., Yang Q., Guo C. (2019). Matrine improves skeletal muscle atrophy by inhibiting E3 ubiquitin ligases and activating the Akt/mTOR/FoxO3α signaling pathway in C2C12 myotubes and mice. Oncol. Rep..

[16] Glass D.J. (2010). Signaling pathways perturbing muscle mass. Curr. Opin. Clin. Nutr. Metab. Care.

[17] Choi S.M., Tucker D.F., Gross D.N., Easton R.M., DiPilato L.M., Dean A.S., Monks B.R., Birnbaum M.J. (2010). Insulin regulates adipocyte lipolysis via an akt-independent signaling pathway. Mol. Cell. Biol..

[18] Fearon K.C., Glass D.J., Guttridge D.C. (2012). Cancer Cachexia: Mediators, Signaling, and Metabolic Pathways. Cell Metab..

[19] The S.N. (2017). A Review on the Medicinal Plant Dalbergia odorifera Species: Phytochemistry and Biological Activity. Evidence-Based Complement. Altern. Med..

[20] Zhao X., Wang C., Meng H., Yu Z., Yang M., Wei J. (2020). Dalbergia odorifera: A review of its traditional uses, phytochemistry, pharmacology, and quality control. J. Ethnopharmacol..

[21] McColl R., Nkosi M., Snyman C., Niesler C. (2016). Analysis and Quantification of in Vitro Myoblast Fusion using the LADD Multiple Stain. BioTechniques.

[22] Setiawan T., Sari I.N., Wijaya Y.T., Julianto N.M., Muhammad J.A., Lee H., Chae J.H., Kwon H.Y. (2023). Cancer cachexia: Molecular mechanisms and treatment strategies. J. Hematol. Oncol..

[23] Webster J.M., Kempen L.J.A.P., Hardy R.S., Langen R.C.J. (2020). Inflammation and Skeletal Muscle Wasting During Cachexia. Front. Physiol..

[24] Katakami N., Uchino J., Yokoyama T., Naito T., Kondo M., Yamada K., Kitajima H., Yoshimori K., Sato K., Saito H. (2018). Anamorelin (ONO-7643) for the treatment of patients with non–small cell lung cancer and cachexia: Results from a randomized, double-blind, placebo-controlled, multicenter study of Japanese patients (ONO-7643-04). Cancer.

[25] Garcia J.M., Friend J., Allen S. (2013). Therapeutic potential of anamorelin, a novel, oral ghrelin mimetic, in patients with cancer-related cachexia: A multicenter, randomized, double-blind, crossover, pilot study. Support. Care Cancer.

[26] Hanada K., Fukasawa K., Hinata H., Imai S., Takayama K., Hirai H., Ohfusa R., Hayashi Y., Itoh F. (2022). Combination therapy with anamorelin and a myostatin inhibitor is advantageous for cancer cachexia in a mouse model. Cancer Sci..

[27] Wakabayashi H., Arai H., Inui A. (2021). The regulatory approval of anamorelin for treatment of cachexia in patients with non-small cell lung cancer, gastric cancer, pancreatic cancer, and colorectal cancer in Japan: Facts and numbers. J. Cachexia Sarcopenia Muscle.

[28] Wang Y., Sun X., Yang Q., Guo C. (2023). Cucurbitacin IIb attenuates cancer cachexia induced skeletal muscle atrophy by regulating the IL-6/STAT3/FoxO signaling pathway. Phytother. Res..

[29] Liu L., Hu R., You H., Li J., Liu Y., Li Q., Wu X., Huang J., Cai X., Wang M. (2021). Formononetin ameliorates muscle atrophy by regulating myostatin-mediated PI3K/Akt/FoxO3a pathway and satellite cell function in chronic kidney disease. J. Cell. Mol. Med..

[30] Stitt T.N., Drujan D., Clarke B.A., Panaro F., Timofeyva Y., Kline W.O., Gonzalez M., Yancopoulos G.D., Glass D.J. (2004). The IGF-1/PI3K/Akt pathway prevents expression of muscle atrophy-induced ubiquitin ligases by inhibiting FOXO transcription factors. Mol. Cell.

[31] Savova M.S., Mihaylova L.V., Tews D., Wabitsch M., Georgiev M.I. (2023). Targeting PI3K/AKT signaling pathway in obesity. Biomed. Pharmacother..

[32] Vaitkus J.A., Celi F.S. (2017). The role of adipose tissue in cancer-associated cachexia. Exp. Biol. Med..

[33] Yecies J.L., Zhang H.H., Menon S., Liu S., Yecies D., Lipovsky A.I., Gorgun C., Kwiatkowski D.J., Hotamisligil G.S., Lee C.-H. (2011). Akt stimulates hepatic srebp1c and lipogenesis through parallel mtorc1-dependent and independent pathways. Cell Metab..

[34] Guo Z., Cheng X., Feng X., Zhao K., Zhang M., Yao R., Chen Y., Wang Y., Hao H., Wang Z. (2019). The mTORC1/4EBP1/PPARγ Axis Mediates Insulin-Induced Lipogenesis by Regulating Lipogenic Gene Expression in Bovine Mammary Epithelial Cells. J. Agric. Food Chem..

[35] Bing C., Russell S., Becket E., Pope M., Tisdale M.J., Trayhurn P., Jenkins J.R. (2006). Adipose atrophy in cancer cachexia: Morphologic and molecular analysis of adipose tissue in tumour-bearing mice. Br. J. Cancer.

[36] Daas S.I., Rizeq B.R., Nasrallah G.K. (2019). Adipose tissue dysfunction in cancer cachexia. J. Cell. Physiol..

[37] Song G., Park W.Y., Jiao W., Park J.Y., Jung S.J., Ma S., Lee J., Lee K.Y., Choe S.-K., Park J. (2024). Moderating AKT signaling with baicalein protects against weight loss by preventing muscle atrophy in a cachexia model caused by CT26 colon cancer. Biochim. Biophys. Acta (BBA)—Mol. Cell Res..

[38] Arneson-Wissink P.C., Ducharme A.M., Doles J.D. (2020). A novel transplantable model of lung cancer-associated tissue loss and disrupted muscle regeneration. Skelet. Muscle.

